# Some Medical Aspects of Malingering

**Published:** 1918-08-31

**Authors:** 


### THE SIMULATION OF DISEASE.

### II. Some Medical Aspects of Malingering.

The detection of malingering is a matter full of pitfalls. Most practitioners at some stage of their career have been guilty of dismissing as trivial or imaginary some case which has subsequently proved to be far otherwise; and the greater the physician's experience the less likely will he be to regard any case as one of malingering without exhaustive clinical examination. The ruses adopted by the malingerer are so varied and depend so much upon adventitious circumstances that there is little point in enumerating even the more common of them. One example, taken from Sir John Collie's work on the subject, only will suffice to show how constantly the medical man should be on his guard. A patient presented himself, saying that he had had his life insured but had allowed the premiums to lapse. The company had, however, agreed to continue his policy on payment of a cheque for the arrears and a medical certificate that the man was still in good health. The doctor in question expressed his willingness to provide a certificate, but insisted on a physical examination. After some demur on the part of the patient, who explained that it was purely a matter of form and that the fee allowed by the company was a very small one, this was agreed to. Indeed, the examination was done with some thoroughness, the patient stripped to the waist and a careful examination of the heart and lungs made. While thus undressed, placing his hands on his hips, or in that neighbourhood, he struck an attitude, saying with some emphasis: "Now, doctor, are you satisfied? Am I not a fine fellow?" Six months afterwards an insurance company communicated with the doctor, asking why he had given a certificate of good health to one of their assured who had just died of cancer of the rectum, and who had had an inguinal colotomy performed antecedent to the date of his examination. The brave words, the dramatic attitude, the hands on the hips, were all a piece of consummate acting, designed to cover the tell-tale scar of a colotomy wound.

### The Method of Examination.

The mistake too often made of assuming a frankly hostile and suspicious demeanour towards the patient is one that defeats its own ends. Not only does it suggest bias on the part of the examiner, but it at once puts a rogue upon his guard. If the patient is left to tell his own tale, uninterrupted and without leading questions, he is tempted to pile symptom upon symptom, involving himself in a tissue of absurdities and exaggerations. Moreover, a nervous patient who is genuinely ill may be so perturbed by the physician's attitude towards him that he may have every appearance of disingenuousness. Another fatal error is to suggest symptoms by the questions put or the method of examination. If the question "Do you sweat profusely at night?" is asked in that form, an affirmative answer is naturally to be expected. Or if a case of alleged sciatica is under suspicion, the physician should not indicate by his palpation the course of the nerve. A malingerer who came under the writer's notice betrayed himself very neatly by complaining of violent sciatica in *both* legs, thus hoping to emphasise the gravity of his condition. (Sciatica is never bilateral.) Apart from an actual examination, which should, of course, always be made, a very shrewd guess can often be made from the patient's own story and his manner of telling it. The really sick man describes his sufferings with details not often known to the layman who has not experienced them. He looks ill. If a joint is affected and he complains of stiffness and immobility, there will almost certainly be wasting of the muscles around that joint.

### The Problem of Hysteria.

But by far the thorniest problem that the expert has to face is the differential diagnosis between hysteria and simulation. In hysteria we have primarily an exaggeration of the normal imitative impulse, or, as Oppenheim puts it, "an exaggeration of the normal psychical reactions." The emotional threshold is lowered, painful stimuli are more readily perceived, the behaviour becomes fantastic, exaggerated, and illogical. A point of the utmost importance is the ready suggestibility of the patient, both from without and by auto-suggestion, and the abnormal persistence of such abnormal impressions and ideas. Of especial importance is this in dealing with cases in which a claim for compensation is made by a hysterical individual after some trifling injury. The hysteric can reproduce with vivid intensity past painful impressions; in the same way a slight feeling of discomfort becomes magnified into a "horrible" pain, although the appearance of the patient belies any statement of genuine suffering. Argument is of no avail in persuading the patient of the imaginary nature of his woes. His conclusions are not based upon reason, and reason is powerless to displace his *idée fixe*.

Thus a hysterical girl, in a case quoted by Janet, who showed persistent and extreme contraction of the field of vision, was yet able to play tennis with more than usual skill, although had the disease been organic she would have been scarcely able to walk. How like, again, to malingering are those cases of hysterical anæsthesia in which the patient affirms that she cannot feel the lightest touch, and yet with her eyes closed says "No!" to every isolated contact with the pencil or finger. The consciousness of untruth is obvious, and yet the attitude of the patient is a perfectly genuine one.

### Some Points of Distinction.

It is clear that any case may present symptoms due to (*a*) organic disease, (*b*) hysteria, (*c*) simulation; or of $(a+b)$ or $(b+c)$. The bulk of the symptoms or signs formerly deemed pathognomonic of

☛ The previous article appeared on August 24, p. 449.

**The Simulation of Disease—(*continued*).**

organic disease—fever, anuria, ankle clonus, etc.—have been counterfeited in hysteria. Indeed, the mimicry, although involuntary, may be so exact as to deceive the most practised observer, in contrast to the malingerer whose counterfeit is but a clumsy parody. The sheet anchor of diagnosis is *the disparity between subjective complaints and objective changes*, the only exception being *anorexia nervosa*, which may lead to death from starvation. In the nervous system certain signs are simulated so rarely as to be practically diagnostic of organic change. Such are Babinski's plantar reflex, nystagmus (though in certain cases it can be reproduced at will), optic neuritis and atrophy, degenerative atrophy of certain muscles or muscle groups, incontinence of urine or fæces, anæsthesia corresponding more or less exactly with the distribution of a given nerve trunk. There may be disparity between the symptoms presented; thus a patient may have paraplegia but no loss of sphincter control, hemianæsthesia without hemiplegia etc. There may be positive evidence of hysteria, *globus*, concentric constriction of the visual fields, fits, and the like.

Between hysteria and malingering a few points aid in diagnosis. The deceit of the hysteric is unconscious, and he courts rather than avoids examination. Design is less obvious, and he readily commits himself to inconsistencies; when charged with a misstatement he grows the more emphatic, his passion is perfectly genuine, and not morose or embarrassed. Again, when not under observation the hysteric still continues—let us say, his peculiarities of gait, although he is apt to exaggerate them under the stimulating effect of an audience. The malingerer readily drops his deception when the need for it has passed.

There is no royal road to diagnosis in doubtful cases, and a little worldly wisdom is worth a great deal of theoretical knowledge.

### Traumatic Lumbago.

The shirker is most prone to complain of pains of a nature necessarily difficult to verify. Thus lumbago or lumbar myalgia—partly, no doubt, because the medical profession have been all too ready in the past to lump together all cases of vague backache, partly because of the extreme difficulty of refuting the patients' statements on this score—is a fruitful source of litigation under the Workmen's Compensation Act.

The first thing to note in a suspicious case is the patient's gait. Is there rigidity of the lumbar spine? Is the walk cautious or groping? Does he sit down gingerly and when rising place his hands on his thighs to gain support?

In the physical examination, note whether the normal lordotic curve is abolished when the patient bends down, and whether the limits of backward and forward dorsiflexion are diminished. If the lumbar curve entirely disappears in the arching of the back the case is undoubtedly one of malingering. Note, too, the exact localisation of the painful spot. In a traumatic lumbago the pain is well defined and almost invariably unilateral. In a simulated case the patient cannot locate it with exactness, and if his attention be distracted, firm pressure can be made at the alleged painful spot without complaint. In doubtful cases the pulse rate and pupil reaction can be noted whilst pressure is applied, points of great value in estimating the effect of painful stimuli. If the diagnosis is still doubtful, a skiagram is of the greatest value. Many cases of traumatic lumbago have been treated as rheumatism or gout till the *x*-ray revealed a fracture of the transverse processes of the lumbar vertebræ.

### The Private Practitioner's Difficulty.

It has been impossible within the scope of two brief articles to do more than touch upon a few salient points in the study of malingering. The vast field of war injuries has been purposely omitted. The would-be malingerer, in these days of travelling boards and elaborate history sheets, stands little chance of successful dissimulation. Nor is there any reason to believe that it is practised to any great extent among the troops. The problem is a simple one where expert advice is readily available. The cases of real difficulty are those where the private practitioner is called upon to pass judgment on a case of simulated nervous disease where an adverse verdict, unless capable of ample proof, is likely to reflect in no very favourable light upon his own professional abilities.

### Want of Direct Evidence.

The manifestations of nervous disorder are so protean that in any case of real doubt expert testimony is essential. In panel work the practitioner is severely handicapped by the absence of any *dossier* of previous diseases and family history. Only too often he is obliged to accept the patient's unqualified statements against his better judgment for want of direct evidence to the contrary. A systematic medical examination of every insured person at the age of sixteen would provide some sort of foundation upon which to build a diagnosis. A similar overhauling every year represents an attainable ideal. But it is well to remember that a knowledge of mankind, coupled with tact and sympathy, are often of the greatest service in persuading a misguided patient of the error of his or her ways. To restore efficiency and not to secure conviction should always be the first aim of the physician.

